# Lipase-Catalyzed Synthesis of Sucrose Monolaurate and Its Antibacterial Property and Mode of Action against Four Pathogenic Bacteria

**DOI:** 10.3390/molecules23051118

**Published:** 2018-05-08

**Authors:** Shi-Yin Shao, Yu-Gang Shi, Yu Wu, Li-Qing Bian, Yun-Jie Zhu, Xin-Ying Huang, Ying Pan, Lu-Yao Zeng, Run-Run Zhang

**Affiliations:** 1 Zhejiang Provincial Collaborative Innovation Center of Food Safety and Nutrition, Zhejiang Gongshang University, Hangzhou 310035, China; 16030000214@pop.zjgsu.edu.cn (S.-Y.S.); wuyu6939@sina.com (Y.W.); 16030000222@pop.zjgsu.edu.cn (L.-Q.B.); 18363851057@163.com (Y.-J.Z.); elandzhou@163.com (X.-Y.H.); Holalailai@163.com (Y.P.); zengluyao159@163.com (L.-Y.Z.); m17826852919_1@163.com (R.-R.Z.); 2 School of Food Science and Biotechnology, Zhejiang Gongshang University, Hangzhou 310035, China

**Keywords:** ionic liquids, lipase, biocatalysis, sucrose monolaurate, antimicrobial activity, biofilm

## Abstract

The aim of this work was to evaluate the antibacterial activities and mode of action of sucrose monolaurate (SML) with a desirable purity, synthesized by Lipozyme TL IM-mediated transesterification in the novel ionic liquid, against four pathogenic bacteria including *L. monocytogenes, B. subtilis, S. aureus*, and *E. coli.* The antibacterial activity was determined by minimum inhibitory concentration (MIC), minimum bactericidal concentration (MBC), and the time–kill assay. SML showed varying antibacterial activity against tested bacteria with MICs and MBCs of 2.5 and 20 mM for *L. monocytogenes,* 2.5 and 20 mM for *B. subtilis,* 10 and 40 mM for *S. aureus*, respectively. No dramatic inhibition was observed for *E. coli* at 80 mM SML. Mechanism of bacterial inactivation caused by SML was revealed through comprehensive factors including cell morphology, cellular lysis, membrane permeability, K^+^ leakage, zeta potential, intracellular enzyme, and DNA assay. Results demonstrated that bacterial inactivation against Gram-positive bacteria was primarily induced by the pronounced damage to the cell membrane integrity. SML may interact with cytoplasmic membrane to disturb the regulation system of peptidoglycan hydrolase activities to degrade the peptidoglycan layer and form a hole in the layer. Then, the inside cytoplasmic membrane was blown out due to turgor pressure and the cytoplasmic materials inside leaked out. Leakage of intracellular enzyme to the supernatants implied that the cell membrane permeability was compromised. Consequently, the release of K^+^ from the cytosol lead to the alterations of the zeta potential of cells, which would disturb the subcellular localization of some proteins, and thereby causing bacterial inactivation. Moreover, remarkable interaction with DNA was also observed. SML at sub-MIC inhibited biofilm formation by these bacteria.

## 1. Introduction

Sugar fatty acid esters (SFAE) are diverse compounds consisted of a hydrophilic carbohydrate moiety and one or more (saturated or unsaturated) fatty acids as lipophilic moieties, which are widely used as emulsifiers in food industry due to their high biodegradability and safety. SFAE are synthesized by chemical and enzymatic methods. However, many consumers are concerned about the potential adverse side effects of synthetic chemicals in food. Thus, it is of vital importance to develop the novel ways of biotransformation with many advantages (mild reaction conditions, exclusive chemo-, region-, and stereoselectivity; high atom economy; and limited waste), compared to traditional chemical synthesis. However, a major challenge in the enzymatic synthesis of SFAE is the selection of appropriate solvent system to dissolve substrates with different physical property (polar sugars and non-polar fatty acid). Some hydrophilic organic solvents (e.g., dimethyl sulfoxide) are able to dissolve both sugars and fatty acids at relatively high concentration, but the activity of most enzymes are retarded in these conventional solvents. Ionic liquids (ILs) are considered as an environmentally friendly alternative to organic solvents and carry numerous intriguing properties that are suitable for numerous applications in biotransformation [1]. The benefits of ILs to biocatalysis include increased stability or protective effects for enzymes, adaptable solubility properties, convinced impact on the specificity of biocatalysts or the shift in equilibrium of the reaction, and recyclability [2,3]. In our laboratory, we have been developing new functionalized ILs in parallel with evaluating them as novel media for biological applications [4,5]. Therefore, we pursued to explore whether the new IL is also suitable for the enzymatic synthesis of SFAE.

Among SFAE, sucrose monolaurate (6-*O*-lauroylsucrose, SML) is considered the potent inhibitor of microbial activity and their properties are being actively explored, yet there are conflicting data on the microbes effected. 500 and 1000 µg mL^−1^ SML showed inhibition against *Escherichia coli* O157:H7 on the surface of raw sliced beef [6]. 1% SML effectively inhibited the growth of *Zygosaccharomyces bailii* and *Lactobacillus fructivorans* in salad dressings during storage at 27 °C [7]. 1000 µg mL^−1^ SML with 600 µg mL^−1^ EDTA caused a 3.0 log reduction of *Yersinia enterocolitica* on egg shell surface [8]. 200 μg mL^−1^ SML inhibited the growth of *E. coli* [9]. SML at 4 mg mL^−1^ inhibited *Bacillus* sp. and *E. coli* [10]. Zhao et al. [11] reported SML showed antibacterial activities against five pathogenic bacteria including *E. coli*. However, Conley and Kabara [12] reported that 125 μg mL^−1^ commercial sucrose laurate (58%, SML) showed no inhibition of *E. coli*. Monk et al. [6] observed that the growth of *E. coli* was unaffected in TSB containing 1 mg mL^−1^ SML. Enzymatically synthesized SML (6.25 mg mL^−1^) resulted in no decrease in the growth of *E. coli* during 28 h [13]. Gram-negative bacteria (*E. coli* O157:H7, *Klebsiella pneumoniae*, *Salmonella enterica serotype Typhimurium*) did not show a change in growth in SML compared to the controls [14]. Although the antibacterial activities of SFAE have been extensively studied, the information related to particularly SML, especially with high purity, has not been well defined. In addition, the sucrose esters preparation that was used normally contained small amounts of di- and triester forms, so it is hard to evaluate how the ester forms contribute to the overall antibacterial activity. To the best of our knowledge, little is known about the mode of action or antibacterial mechanism of SML against pathogenic bacteria.

In the present report, firstly, we had developed the enzymatic synthesis SML by lipozyme TL IM-mediated transesterification in a novel functionalized ionic liquid. Then, in order to ascertain the antibacterial efficacy and potential mechanism of SML against four pathogenic bacteria including *Listeria monocytogenes* (*L. monocytogenes*), *Bacillus subtilis* (*B. subtilis*), *Staphylococcus aureus* (*S. aureus*), and *Escherichia coli* (*E. coli*), the in vitro antibacterial activity of SML with high purity (more than 94%) were evaluated by measuring MIC and MBC values, as well as kill-time assays. Moreover, the antibacterial mechanisms towards representative bacteria were determined by cell morphology, cellular lysis, membrane permeability, K^+^ and β-galactosidase leakage, zeta potential and cellular DNA, as well as biofilm inhibition.

## 2. Materials and Methods

### 2.1. Materials

Sucrose and vinyl laurate with >98% purity were purchased from Sigma-Aldrich (Shanghai, China). Sucrose monolaurate (≥97%) was obtained from Sigma-Aldrich (Shanghai, China). Lipozyme TL IM (*T. lanuginosus* lipase immobilized on silica gel) was a gift from Novo Nordisk (Copenhagen, Denmark). 3 Å and 4 Å Molecular sieves were purchased from Sinopham Chemical Reagent Co., Ltd. (Shanghai, China). Ionic liquid, 3CIM(EO)][NTf_2_] (1-(2-(2-methoxyethoxy)ethyl)-5,6,7,8-tetrahydroimidazo[1,2-a]pyridin-1-ium bis(trifluoromethylsulfony)imide) (99%, HPLC) was synthesized and purified in our laboratory. All other reagents were of analytical grade. All other chemicals of analytical or HPLC grade were purchased from commercial sources in China. Vinyl laurate and other organic solvents were stored over 4 Å molecular sieves, at least 24 h prior to use.

### 2.2. Lipase-Catalyzed Transesterification for SML in Ionic Liquid

#### 2.2.1. Synthesis of [3CIM(EO)][NTf_2_]

The procedure of synthesis was conducted according to our method [5] with minor modifications. Methanesulfonyl chloride (36 mmol, in 30 mL DCM) was added to an 50 mL DCM solution of 2-(2-methoxyethoxy) ethanol (22 mmol) and triehylamine (56 mmol) at 0 °C. The contents were stirred in an ice bath for 1 h. Then, the reaction was washed with 0.5 M citric acid (3 × 20 mL) and 1 M sodium bicarbonate (3 × 20 mL). The organic layer was dried over anhydrous Na_2_SO_4_ and evaporated under reduced pressure to obtain colorless liquid 2-(2-methoxyethoxy) ethyl methanesulfonate. 6,7-Dihydro-5*H*-pyrrolo[1,2-a] imidazole (10 mmol) was added into a round-bottomed flask containing 2-(2-methoxyethoxy) ethyl methanesulfonate. The reaction was conducted at 80 °C overnight. After cooling to the room temperature, the whole mixture was subsequently washed with ether (3 × 10 mL) to give a light yellow liquid, methanesulfonate salt, (65–96% yield). To a 150 mL round-bottomed flask was then added methanesulfonate salt (2.5 g) followed by the addition of bistrifluoromethanesulfonimide lithium salt (1:1 equiv) and water (5 mL). The reaction was allowed to proceed the ion exchange for 12 h at room temperature. The reaction was extracted with DCM (3 × 5 mL) and dried over anhydrous Na_2_SO_4_ and concentrated to obtain the colorless liquid [3CIM(EO)][NTf_2_] with good isolated yield (70–90%).

#### 2.2.2. General Procedure of the Lipase-Catalyzed Transesterification in the Ionic Liquid

Crystalline sucrose was ground manually to powder in a porcelain mortar, sieved through a mesh with a pore size of 0.2 mm, dried in a 60 °C oven generally overnight and then stored in desiccators. Lipase-catalyzed synthesis of SML with vinyl laurate in the mixtures of ionic liquid-organic solvent ([3CIM(EO)][NTf_2_] and 2-methyl-2-butanol (2M2B)) (1.5:1, *v*/*v*) was carried out in a glass vial with Teflon caps to avoid the escape of volatile organic compounds, especially for organic solvents. Sucrose (45 mM) and vinyl laurate (180 mM) were mixed with the media mentioned above. 50 g/L of enzyme and 3 Å molecular sieve (10%, *w*/*v*) was put into the mixture. The reaction was allowed to proceed at 60 °C for 72 h. After centrifugation, TLC and HPLC technic was used to analyze the supernatants. The sucrose esters were eluted with ethyl acetate: methanol: H_2_O (17:2:1 *v*/*v*/*v*). The purification was monitored by TLC. The fractions containing SML were pooled and the solvent was evaporated to obtain SML for further study.

Position of the fatty acid in the sucrose and monoacylation were confirmed by NMR spectroscopy. ^1^H- and ^13^C-NMR-spectra were recorded on a Bruker Avance 500 spectrometer (Bruker Instruments, Karlsruhe, Germany).

*Sucrose Monolaurate (SML)*. *6-O-Lauroylsucrose*. solid, ^1^H-NMR (500 MHz, DMSO): *δ* 5.18 (d, *J* = 3.6 Hz, 1H); 4.34–4.10 (m, 2H); 4.02 (dd, *J* = 11.7, 6.0 Hz, 1H); 3.99–3.81 (m, 2H); 3.71 (ddd, *J* = 18.6, 13.7, 13.0 Hz, 2H); 3.63–3.44 (m, 6H); 3.44–3.24 (m, 4H); 3.18 (ddd, *J* = 12.5, 9.9, 3.2 Hz, 2H); 3.06 (d, *J* = 9.4 Hz, 1H); 2.37–2.12 (m, 2H); 1.63–1.36 (m, 2H), 1.24 (s, 16H); 0.86 (t, *J* = 6.8 Hz, 3H). ^13^C-NMR: *δ* 173.47 (7–C=O), 104.33 (2’–C), 91.92 (1–C–O), 83.18 (5’–C), 77.39 (3’–C–OH), 74.94 (4’–C), 73.13 (3–C), 71.98 (5–C), 70.61 (2–C), 70.48 (4–C), 64.03 (6–C), 63.07 (6’–C), 62.64 (1’–C), 33.80 (–CH_2_– Chain), 31.76(–CH_2_– Chain), 29.47 (–CH_2_– Chain), 29.39 (–CH_2_– Chain), 29.18 (–CH_2_– Chain), 28.97 (–CH_2_– Chain), 24.88 (–CH_2_– Chain), 22.56 (–CH_2_– Chain), 14.43 (18–CH_3_). ESI-MS: [M + H_2_O]^+^ = 542.316.

#### 2.2.3. Analytical Procedure

Reaction were examined by TLC on Silica gel 60 F254 aluminum sheets using chloroform: methanol: acetic acid: H_2_O (75:15:5:3 *v*/*v*/*v*/*v*) as the eluting system. The compounds were colored by spraying a color agent with *p*-anisaldehyde–aceticacid–95% ethanol–sulfuric acid (12.5:2.5:225:12.5 *v*/*v*/*v*/*v*) and visualized by heating at 105 °C for 15 min. HPLC analysis was performed by HPLC using a Waters pump (Waters 1525, Waters, Sutton, MA, USA) with evaporative light scattering detector (ELSD, Waters 2424). A Purospher RP-18e column (5 × 250 × 4.6 mm^2^, Merck, Darmstadt, Germany) was used, and the mobile phase was a mixture of methanol and water (90:10 *v*/*v*) at a flowrate of 1 mL min^−1^. The yield of SML was calculated from the HPLC data.

### 2.3. Bacterial Strains and Growing Conditions

Four common pathogenic bacterial strains were used in the study. The Gram-positive bacteria, *Listeria monocytogenes* ATCC 19114 (*L. monocytogenes*), *Bacillus subtilis* CMCC(B) 63501 (*B. subtilis*), and *Staphylococcus aureus* CMCC(B) 26003 (*S. aureus*); and the Gram-negative bacteria, *Escherichia coli* CMCC(B) 44102 (*E. coli*) were obtained from National Center For Medical Culture Collections (Beijing, China), and were grown and maintained in Tryptone Soy broth (TSB) and on Trypticase Soy agar (TSA) (Hangzhou Microbial Reagent Co. Ltd, Hangzhou, China). Strains were maintained on TSA slants at 4 °C. A 16-h culture in TSB at 37 °C was diluted with TSB to achieve an inoculum of 10^6^ CFU mL^−1^ approximately. The cells numbers in the suspensions was identified by duplicate plating from 10-fold serial dilution on TSA and counting the colonies after incubation at 37 °C for 24 h.

### 2.4. Antimicrobial Activity

#### 2.4.1. Minimum Inhibitory Concentration (MIC) and Minimum Bactericide Concentration (MBC)

MIC and MBC were determined by broth macrodilution assay with some modification [15]. Serial two-fold dilutions of SML were prepared in TSB at concentrations of 1.25, 2.5, 5, 10, 20, 40, and 80 mM. One tube with the same volume of TSB without SML was set as the negative control. Inocula were added into all the tubes to achieve the initial inoculum of 10^6^ CFU mL^−1^ approximately. All tubes were incubated at 37 °C for 24 h, a 1 mL portion was removed from 10 mL cell suspensions in each tube for colony counting by decimal dilution with sodium chloride solution (0.85%, *w*/*v*) , and plated out onto TSA. Each concentration was assayed in triplicate. The MIC was defined as the lowest concentration resulting in maintenance or reduction of inoculums viability for all the triplicates compared to the negative control after 24 h. The minimum bactericidal concentration (MBC) is defined as the concentration where nearly 99% or more of the initial inoculums are killed after 24 h.

#### 2.4.2. Growth Curve and Time–Kill Kinetics Analysis

Briefly, Indicators were cultured to exponential phase (OD_600_ = 0.5–0.7) at 37 °C and 125 µL of the culture was transferred into each well on 96-well microtiter plates. SML was dissolved in the cultures to obtain final concentrations of 1× MIC, 2× MIC, and 4× MIC, and samples without SML were used as control. Bacteria were further cultured at 37 °C, and cell growth was monitored by enzyme micro-plate reader at 600 nm at 0.5 h intervals, using a multimode plate reader, and lysis was observed.

The time–kill test was performed to determine the killing kinetics of SML against tested bacteria [16]. Individual bottles containing SML broth dilutions with various concentrations were cultured with the inoculum with a density of 10^6^ CFU mL^−1^ at 37 °C with shaking at 180 rpm. After 0, 2, 4, 8, 12, 20, 24, and 48 h of incubation, aliquots of 1 mL was removed from each tube for colony counting by decimal dilution in 0.85% (*w*/*v*) sodium chloride solution, and plated out onto TSA at 37 °C for 12–18 h, and the number of survivors (CFU mL^−1^) was determined by counting the colonies. Time–kill curves were constructed by plotting the Log CFU mL^−1^ versus time. The theoretical detection limit was 10 CFU mL^−1^, corresponding to 1 Log CFU mL^−1^.

### 2.5. Antibacterial Mechanism

#### 2.5.1. Propidium Iodide Uptake Test

The fluorescent dye propidium iodide (PI; alladin, P113815) was used to evaluate cell membrane damage of bacteria. The PI uptake test was conducted according to the method with some modifications [17]. For each bacterium, after inoculation, all the solutions were incubated at 37 °C under shaking conditions (180 rpm) for 24 h. A 5 mL portion from each tube was removed and then centrifuged at 6000× *g* at 4 °C for 15 min. The supernatants were discarded and cells were washed three times with PBS (0.1 mmol L^−1^, pH 7.2) and then cell pellets were resuspended in 10 mL PBS (0.1 mmol L^−1^, pH 7.2) with the final cells concentration of 10^6^ CFU mL^−1^. SML was added at the final concentration of 1× MIC and then incubated at 37 °C under shaking conditions (180 rpm) for 4 h. A PI stock solution of 1 mg mL^−1^ was prepared. For evaluation of PI uptake after SML treatment, cells were incubated with 0.3 mL PI solution in the dark at 37 °C for 10 min. Fluorescence was measured at an excitation wavelength of 495 nm and an emission wavelength of 500–700 nm with a 5-nm slit in a fluorimeter (RF-5301PC, Shimadzu, Kyoto, Japan). The parallel sample without SML was used as the negative control. Three independent experiments were performed for each condition tested.

#### 2.5.2. Cell Constituents’ Release

The cell integrity is examined by determining the release of cell constituents into supernatant according to Diao’s method with some modifications [18]. Cells from the working culture of tested bacteria were collected by centrifuged for 10 min at 5000× *g*, washed three times with PBS (0.1 M, pH 7.0), and resuspended in the same buffer. 5 mL of cell suspension were incubated with 1× MIC SML at 37 °C under agitation for 6 h. Then, 2 mL of each sample was collected and centrifuged at 10,000× *g* for 5 min. Negative control groups containing bacterial supernatant without SML treatments were tested similarly. The concentrations of proteins in supernatants were established by Bradford assay [19]. 1 mL of diluted supernatant and 5 mL of Coomassie Brilliant Blue G-250 reagent were carefully mixed and its absorbance was measured at 595 nm. The protein content was determined by using a calibration curve prepared with bovine serum albumin. Reducing sugars in supernatants were determined using the 3,5-dinitrosalicylic acid (DNS) method by taking 1 mL of diluted supernatant along with 1 mL of DNS reagent. A mixture was heated in boiling water for 10 min. After cooling to room temperature, 10 mL of distilled water was added. The absorbance of the reaction mixtures was measured at 540 nm and compared with a glucose calibration curve to obtain the concentration of reducing sugars. The amounts of DNA and RNA released from the cytoplasm in supernatant were analyzed by measuring the absorbance at 260 nm. Potassium ion concentration in TSB in supernatants were determined by the method modified from Zhang’s method [20]. Cultures of bacteria with a density of 10^6^ CFU mL^−1^ were exposed to SML with fixed concentrations in TSB and all the solutions were incubated at 37 °C under shaking conditions (180 rpm). After 0, 0.5, 2, 4, 8, 12, 20, and 24 h of incubation, aliquots of 1 mL was removed from each tube and a cell-free supernatant was harvested by centrifugation at 8000× *g* for 15 min and the suspension was filtered through a millipore filter (Hydrophilic PTFE needle filter, ANPEL Laboratory Technologies Inc., Shanghai, China) with a 0.45-μm nylon membrane and then 0.1 mL filtrate was diluted with 4.9 mL HNO_3_ solution (2%) for measuring the amount of extracellular potassium ions via inductively coupled plasma mass spectrometry (ICP-MS; Agilent 7700×, Agilent Technologies Japan, Tokyo, Japan).

#### 2.5.3. Cytoplasmic β-Galactosidase Released from Cells after SML Treatment

The bacterial suspensions of *L. monocytogenes, S. aureus*, and *E. coli* (1.0 × 10^8^ CFU mL^−1^) were treated with solutions of SML at 20 mM containing 1.0 mM MgCl_2_ and 10 mM KCl at 37 °C with orbital shaking for 2 h. Untreated *L. monocytogenes, S. aureus*, and *E. coli* were used as negative controls, respectively. The samples were then centrifuged at a 4000× *g* for 10 min, then the supernatants were retrieved for use in the colorimetric assay. The supernatants were diluted ten-fold in PBS solution (950 μL), and then 50 μL of 1.0 mM *o*-nitrophenol (ONP) was added to the supernatants and allowed to react at room temperature for 2 h. Then, 200 μL of each sample was transferred into a 96-well flat bottom microplate, and the absorbance of the samples was measured using a monochromatic microplate spectrophotometer at 420 nm.

#### 2.5.4. Evaluation of Zeta Potential

The zeta potential of the bacterial suspensions was determined as previously reported with some modifications [21]. Various bacteria were incubated overnight in TSB at 37 °C at 180 rpm agitation. The cells were centrifuged twice at 5000× *g* and 25 °C for 10 min and washed with sterile distilled water. The OD640 nm of cell suspensions was set to 0.2 ± 0.02. 1.8 mL of this culture was added to 200 μL of SML (to a final concentration of 10 mM) and incubated at 30 °C and 120 rpm for 1 h. A negative control was prepared with sterile distilled water. The zeta potential of the bacterial suspensions was recorded using a Nano Zetasizer (Malvern Instruments), in carefully filled zeta potential cells (DTS1060, Malvern, UK), at the room temperature (20–25 °C).

#### 2.5.5. Effect of SML on Bacterial DNA

Bacteria genomic DNA was extracted by using TIANamp Bacteria DNA Kit (Tiangen Biotech, Co., Ltd., Beijing, China). The purity of the extracted DNA was determined by the optical density method (OD260/OD280 = 1.978), and the concentration of DNA measurement was conducted by using the ultraviolet absorbance at 260 nm (UV 2600, Shimadzu, Kyoto, Japan) at the room temperature. The bacteria DNA and SML were dissolved in 10 mM Tris–HCl buffer (pH 7.2) and then mixed to obtain various DNA/SML samples with increasing SML concentrations (0, 5, and 10 mM). DNA mixed with ethanol was used as negative control because SML was dissolved in ethanol. After incubation at 37 °C for 10 min, absorbance of the mixed solutions was measured in the range of wavelengths 230–350 nm (SpectraMax i3x multi-mode detection platform, Molecular Devices, Wals, Austria). Electrophoresis was performed to separate and visualize DNA fragment in a 0.8% agarose gel at 130 V for 25 min. DNA was stained with the GelRed dye.

#### 2.5.6. Biofilm Formation Assay

Biofilm formation was quantified in regular 96-well microtiter plates according to a previously described method [22] with minor modifications. The overnight-grown cultures were diluted 1:100 in fresh TSB medium containing appropriate concentrations of SML, transferred to sterile 96-well microtiter plates at 200 μL per well and incubated at 37 °C without shaking for 48 h.

#### 2.5.7. Scanning Electron Microscopy (SEM) Analysis

SEM studies were carried out as previously reported with some modifications [23] to determine the efficacy of the SML and the morphological changes of bacteria strains. Bacteria at approximate 10^6^ CFU mL^−1^ were treated with 1× MIC SML. After 20 h of incubation at 37 °C, cells were harvested by centrifugation at 10,000× *g* for 10 min and washed twice with 0.1 M phosphate buffer saline (PBS, pH 7.2), and then fixed with 2.5% (*v*/*v*) glutaraldehyde in PBS (0.1 M, pH 7.0) overnight at 4 °C; washed three times in the PBS (0.1 M, pH 7.0) for 15 min at each step; then postfixed with 1% OsO_4_ in phosphate buffer for 1–2 h and washed three times in the PBS (0.1 M, pH 7.0) for 15 min at each step. After centrifugation, the cells were then dehydrated with an ethanol series (30, 50, 70, 80, 90, 95, and 100%). Cells were dried further by using a Hitachi Model HCP-2 critical point dryer (Hitachi Koki Co. Ltd., Tokyo, Japan) with liquid CO_2_. The dehydrated sample was coated with gold-palladium in Hitachi Model E-1010 ion sputter (Hitachi Koki Co. Ltd., Tokyo, Japan) for 4–5 min and examined under Hitachi Model TM-1000 SEM (Hitachi Koki Co. Ltd., Tokyo, Japan).

### 2.6. Statistical Analysis

All of the experiments were performed in triplicate and the mean ± standard deviation values were reported. The significant differences between the two groups were examined using *t*-test. A *p*-value < 0.05 denoted the presence of a statistically significant difference.

## 3. Results and Discussion

### 3.1. Lipase-Catalyzed Transesterification in Novel Functionalized Ionic Liquid

The yields of the enzymatic synthesis of SML was dependent on many conditions including the type of acyl donor, enzyme, and solvent [14]. In order to gain greater yield, vinyl ester was chosen as the acyl donor as the rate of transesterification of sugar and vinyl ester was much faster than with alkyl esters [24]. Considering both the regioselectivity (6-*O*) and catalytic activity, it had been confirmed that lipase from *T. lanuginosa* (such as Lipozyme TL IM from *Novo Nordisk*) is the most efficient enzyme for this reaction [25]. A conversion of 37% to monolaurate in 36 h was achieved by employing 2M2B as the medium. The resulting monoester was isolated, and the position of acylation determined by ^1^H-NMR, demonstrating that hydroxyl 6-OH was mainly selectively acylated. Furthermore, we found a functionalized ionic liquid ([3CIM(EO)][NTf_2_]) could give a relatively better yield of SML compared to [BMIM][BF_6_] or [BMIM][BF_4_] which were commonly used in biocatalysis to ameliorate the performance of enzymes in conventional organic solvents (no data shown). It has been reported that ILs containing an alkyl ether moiety as a cationic part acted as good media for enzymatic reactions [26]. It is accordingly envisioned that the novel ionic liquid synthesized with the combination of both advantages from the structures of double imidazolium ring and alkyl ether moiety could not only afford a preferred microenvironment for enzymatic reactions with maintaining the relatively high activity of lipase but also improve the solubility of substrates [5]. The addition of [3CIM(EO)][NTf_2_] (50%, *v*/*v*) to the medium gave rise to a notable acceleration of the process, resulting in a 66% conversion to 6-*O*-lauroylsucrose in 24 h. This enhancement of monoester production and shorter reaction time could be related to a better solubilization of sucrose in the medium containing the suitable ionic liquid.

**Scheme 1:**
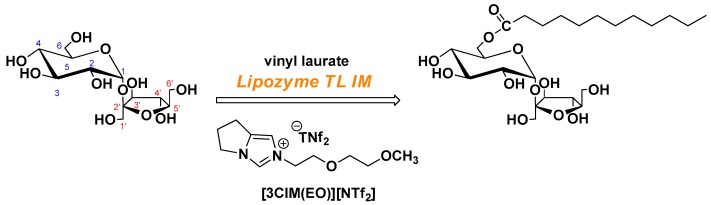
Lipase-catalyzed synthesis of SML in ionic liquid.

In this work, the lipase-catalyzed transesterification of sucrose with vinyl laurate was successfully performed in a binary system consisted of [3CIM(EO)][NTf_2_] and 2M2B (Scheme 1), which proved to be capable of achieving a high SML yield (72%) under the optimum conditions (V_IL_:V_2M2B_ = 1.5:1, sucrose to vinyl laurate molar ratio of 1:3, 60 °C, Lipozyme TL IM concentration of 50 g/L, 24 h). The SML was the main resulting product with small amount of diesters and the purity of SML was higher than 94% after being purified by silica gel column chromatography (Figure S1a). The identity of the ester products was confirmed by NMR analysis and its ^13^C-NMR spectrum showed as Figure S1b, which was shown to be identical to those reported by Potier et al. [27].

### 3.2. MICs and MBCs of SML

Most of the previous studies on antimicrobial properties of SFAE used commercial derivative [6,10]. In this context, SML was obtained as described above and purified to 94% via by silica gel column chromatography. The effect of SML was tested on four different pathogenic bacteria including Gram-positive bacteria (*L. monocytogenes, B. subtilis,* and *S. aureus*) and Gram-negative bacterium (*E. coli*). The bacterial were diluted to 10^6^ CFU mL^−1^ before the addition of SML at final concentrations of 1.25, 2.5, 5, 10, 20, 40, and even 80 mM. The MICs and MBCs of SML against the tested bacteria are shown in Figure S2.

In general, SML showed a higher activity against Gram-positive bacteria than against Gram-negative bacteria. The MICs of SML against Gram-positive bacteria, *L. monocytogenes, B. subtilis*, as well as *S. aureus* were 2.5, 2.5, and 10 mM, respectively. Compared with other tested bacteria, *L. monocytogenes* appear to be the most susceptible to SML, as it shows a significant growth difference from the control at a SML concentration of 2.5 mM. *B. subtilis*, is more sensitive to SML than that of *S. aureus*. The similar results were also obtained by Wagh et al. [28], Habulin et al. [13], and Ferrer et al. [10]. Lactose monolaurate of 2 mM exhibited some growth inhibition against *Listeria* but the MBC was between 5.7 and 9.5 mM [28]. Habulin et al. [13] found the similar effect of SML concentration on the inhibitory of it again *B. cereus*, namely increasing the concentration of SML up to a specific value, 23 mM, resulting in 99% inhibition. On the other hand, enzymatically synthesized SML with the concentration of 7.6 mM could exhibit the maximum percentage of inhibition for *B. subtilis* (95%), while the inhibition could not be found as for *S. aureus* [10]. Moreover, Figure S2 also shows that *L. monocytogenes* and *B. subtilis* has a similar MBC of 20 mM, while *S. aureus* has an MBC of 40 mM.

As for Gram-negative bacteria, *E. coli*, the growth of *E. coli* was slightly less than that of control at concentrations of 40 mM (regarded as the MIC) and only 3% inhibition was observed in the concentration of 80 mM (Figure S2d). The results indicated that Gram-negative bacteria were more resistant to SML than Gram-positive bacteria due to the outer membrane of the Gram-negative bacteria, which restricted diffusion of SML through their lipopolysaccharide covering [29]. Similar results were also found by Monk et al. [6] and Habulin et al. [13]. SML showed no inhibition on the growth of *E. coli* in broth in the concentration of 2 mM [6]. The purified enzymatically synthesized SML in the concentration of 6.25 mg mL^−1^ (12 mM), as well as the commercial SML in concentration of 12.5 mg mL^−1^ caused no decrease in the growth of *E. coli* after 28 h [13]. However, the results are varying in other research groups. Ferrer et al. [10] reported 26% inhibition was obtained at the concentration of 7.6 mM of enzymatically synthesized SML. Kato and Arima [9] reported chemically synthesized SML (58.3%, in purity) at concentrations higher than 0.2 mg mL^−1^ completely inhibited growth, whereas using the same sample from Kato group, no inhibition of Gram-negative organisms including *E. coli* was observed by Conley and Kabara [12]. These discrepancies could be due to the variation in the procedures of preparation and purification for SML, bacteria strains, as well as analysis approaches of antimicrobial activity.

### 3.3. Growth Curve and Time–Kill Analysis

**Figure 1:**
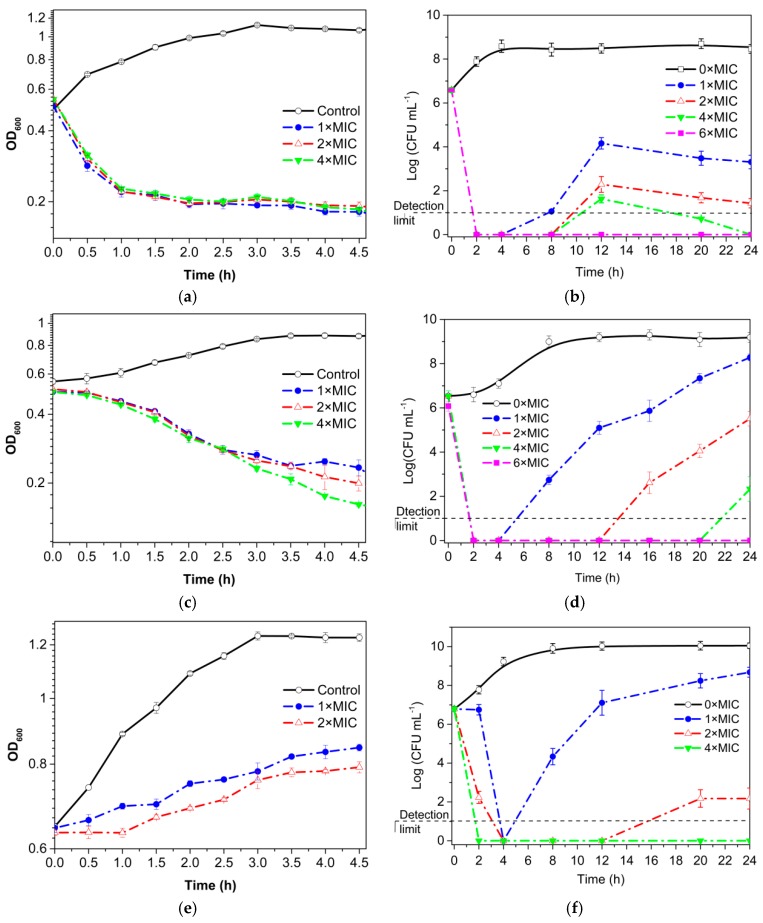

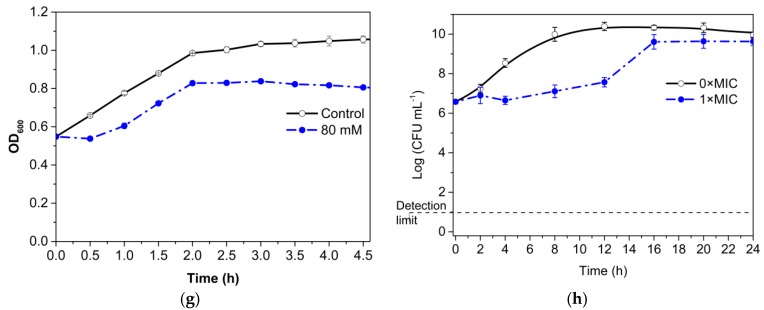
Time–kill curves of SML at the different concentration against various microorganisms. (**a**,**b**) *L. monocytogenes*; (**c**,**d**) *B. subtilis*; (**e**,**f**) *S. aureus*; (**g**,**h**) *E. coli*. The error bars represent the standard deviations.

SML lysed exponentially growing cells of *L. monocytogenes* and *B. subtilis* (Figure 1a,c). The addition of SML to the culture with the final concentration of 1× MIC caused a 72 or 25% reduction in the OD_600_, respectively, after 1 h of incubation. These results suggested that the lysis of *L. monocytogenes* and *B. subtilis* cells induced with SML may be due to the action of autolytic enzymes, as in the lysis by sucrose hexadecanoate described previously [30].

Time–kill assays allow to examine the rate at which different concentrations of enzymatically synthesized SML kill bacteria; the concentration-dependent and time-dependent bactericidal activities of SML can be investigated by this methodology. The results obtained for the time–kill curves are summarized in Figure 1. Time–kill curves differ for each species. SML at higher concentrations led to a more rapid decrease in bacterial number for Gram-positive bacteria. The viability of these Gram-positive cells decreased significantly to 0 Log CFU mL^−1^ after 2 or 4 h of incubation with SML at 1× MIC, compared to nearly 8 Log CFU mL^−1^ of the control. In comparison with the results in Figure 1a–d, the lytic action of SML to *L. monocytogenes* and *B. subtilis* could be ascertained clearly. In addition, during this treatment a great loss of viability occurred that preceded lysis, indicating that autolysis is induced by SML. On the other hand, the viable cells numbers were rapidly declined after 2 or 4 h (Figure 1b,d,f). SML may interact with cytoplasmic membrane (CM) to disturb the regulation system of (a) peptidoglycan (PG) hydrolase(s) activities. Such an enzyme may attack outside PG and the initial partial degradation of PG layer may form rather hole in the layer.

In the treatments with SML at more than 1× MIC concentration, the numbers of viable cells of bacteria were significantly lower than the initial value after 24 h. Above 4× MIC, bactericidal activity promptly was shown in 2 h. Furthermore, the fact that SML at high concentrations showed rapidly bactericidal activity, pointed towards a mechanism of action, infiltration of bacterial membrane. Moreover, when SML concentration was set at 2× MIC, there were 5.16, 1.03, and 4.61 Log CFU mL^−1^ drops in colony counts after 24 h for *L. monocytogenes*, *B. subtilis* and *S. aureus*, respectively (Figure 1b,d,f). Noteworthily, for Gram-positive bacteria, killing was first observed after 2 or 4 h and then the surviving cells regrew, which indicate these cells seemed to adapt to SML. Similar results were also found in bacteria cells treated by other antibacterial compounds, such as sucrose hexadecanoate [30] and amikacin [31].

For the Gram-negative bacteria, *E. coli*, to some extent antibacterial activity was observed before 16 h at 1× MIC SML and then the regrowth occurred (Figure 1d). However, in terms of the time–kill curves, we noted these findings are inconsistent with the results recently reported by Zhao et al. [11], even though the antibacterial agent with similar structure, sucrose monocaprate, was applied to inhibit the growth of *B. cereus, B. subtilis, S. aureus, E. coli*, *and S. typhimurium*.

### 3.4. Permeability of Cell Membrane

**Figure 2:**
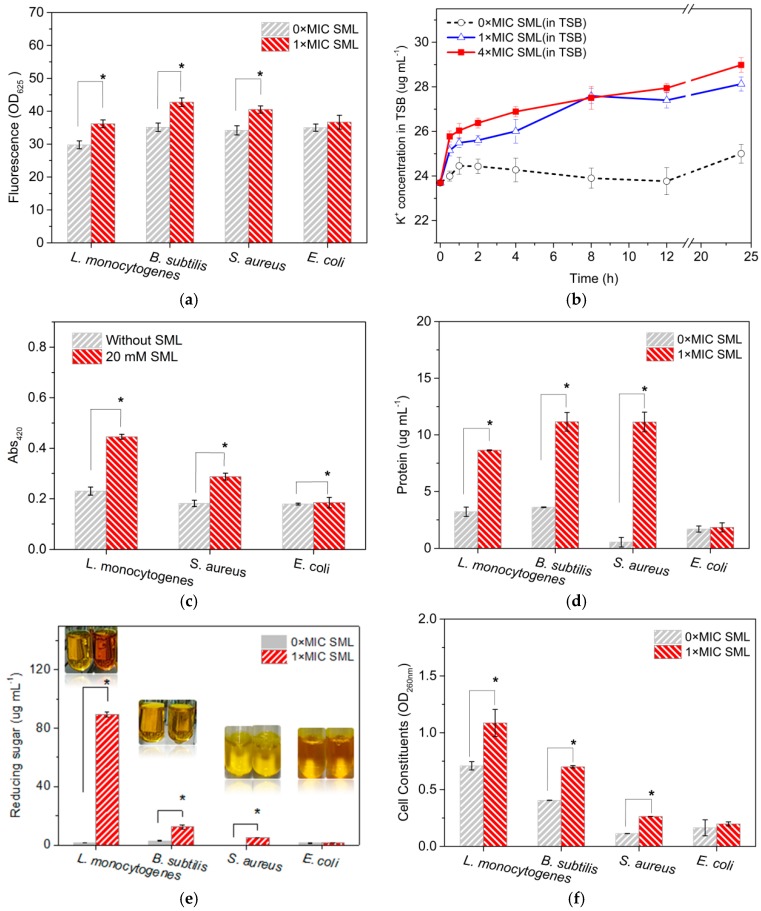
(**a**) Uptake of propidium iodide of various microorganisms after SML treatment. Effect of SML on cell constituents’ release of various bacteria; (**b**) Potassium ion efflux of *L. monocytogenes* cell suspensions in TSB; (**c**) Absorbance of *o*-nitrophenol (ONP) at 420 nm (Abs_420_) from the supernatants of different cells with 20 mM SML treatment; (**d**) Protein. The error bars represent the standard deviations, and the asterisks indicate significant difference between each other; (**e**) UV-absorbing substances leaking (OD_260_ nm); (**f**) Reducing sugar. The error bars represent the standard deviations, and the asterisks indicate significant difference between each other.

**Figure 3:**
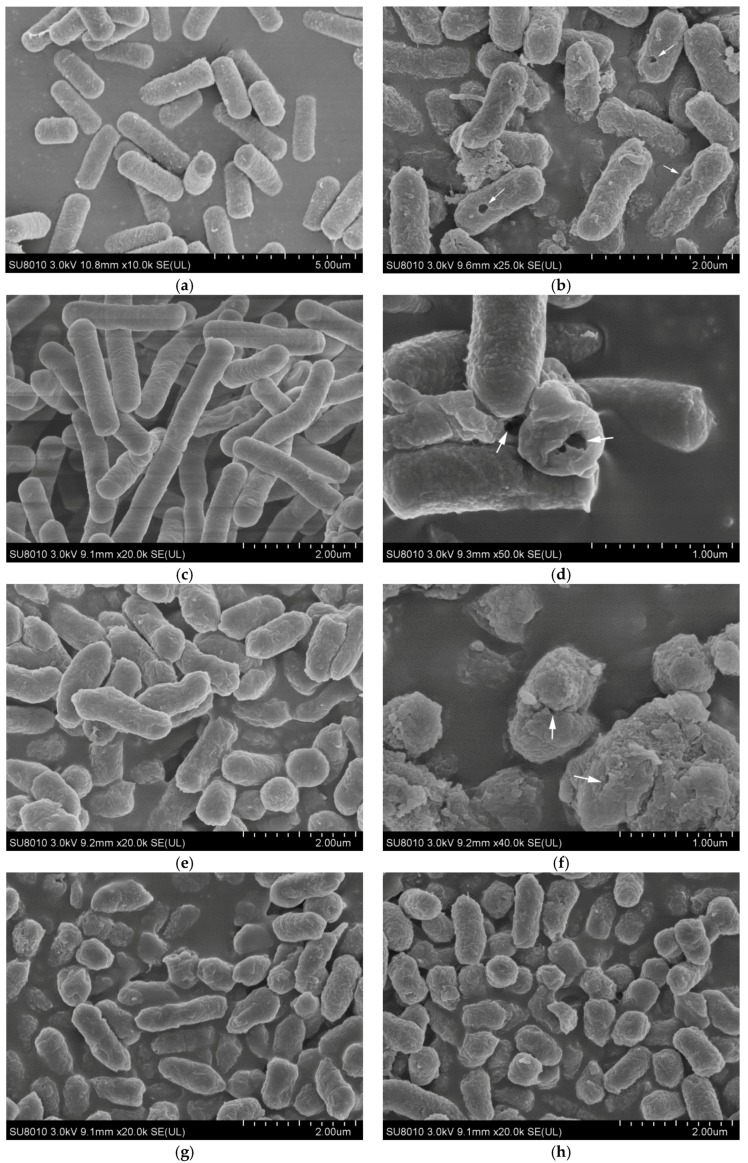
(**a**) SEM of *L. monocytogenes* control (magnification, ×10,000); (**b**) SEM of *L. monocytogenes* after treatment with 1× MIC SML for 24 h (magnification, ×25,000); (**c**) SEM of *B. subtilis* control (magnification, ×20,000); (**d**) SEM of *B. subtilis* after treatment with 1× MIC SML for 24 h (magnification, ×50,000); (**e**) SEM of *S. aureus* control (magnification, ×20,000); (**f**) SEM of *S. aureus* after treatment with 1× MIC SML for 24 h (magnification, ×40,000); (**g**) SEM of *E. coli* control (magnification, ×20,000); (**h**) SEM of *E. coli* after treatment with 1× MIC SML for 24 h (magnification, ×25,000). White arrows indicate the pore-like lesions.

PI uptake, which is intimately related to pore formation in the cell membrane [17]. Cells were treated with SML at 1× MIC for 24 h. Under these conditions PI uptake values varied with type of bacteria (Figure S3 and Figure 2a). The differences in fluorescence intensity (a.u.) caused by PI-DNA interaction could quantitate the strength of CM permeabilization following the SML-induced membrane leakage. Except *E. coli*, PI uptake values for bacteria pretreated with SML at 1× MIC for 24 h were significantly higher than those treated without SML in the present study, which indicate that pores can form in the cell membrane when SML was applied to these bacteria. It also can be observed in SEM analysis (Figure 3b,d,f). In addition, pore formation is dependent on SML concentrations, and a critical concentration is needed for pore formation to lead to membrane destruction (no data shown). In terms of *E. coli* cells, the fluorescence intensity of PI showed no increase compared with the control (*p* > 0.05), indicating that the integrity of *E. coli* cells could not be compromised by 1× MIC SML, which also could be directly observed by SEM (Figure 3g,h).

In parallel with the hole formation, the inside CM could be blown out first due to turgor pressure from the cytoplasm of Gram-positive bacteria to form a bleb. Then, the bleb was broken and inside cytoplasmic materials leaks out. The turgor force for *Bacillus subtilis* is considerable, and up to 20 atm [32]. Therefore, the overall data propose that SML reinforced damage on the bacterial membranes, with remarkable effects on Gram-positive bacteria. Thus, one can propose that SML may act as the antimicrobial agent that weaken the outer membrane of the cells, inducing changes on its permeability. To investigate the effect of SML on cytoplasmic membrane, the permeability of the membranes to intracellular K^+^ and β-galactosidase was also assessed. Figure 2b showed K^+^ efflux from *L. monocytogenes* treated with SML at 1× MIC and 4× MIC for different period of time. In the presence of SML, the K^+^ efflux significantly accelerated for the first 2 h and then continued to increase for the rest of observation time. As shown in Figure 2c, released cytoplasmic β-galactosidase from *L. monocytogenes*, *S. aureus*, and *E. coli* exhibits dose dependence with treatment using SML. Compared to the control, the leakage of cytoplasmic β-galactosidase from *L. monocytogenes* and *S. aureus* treated with SML increased by 1.9 and 1.5 times, respectively. However, in case of *E. coli*, no obvious increase in the leakage of cytoplasmic β-galactosidase was observed, compared to the control.

As a damaged bacterial cell membrane is more permeable, large molecules, such as protein and genetic nucleotide materials leach out from the cells. Compared to the control, the leakage of proteins from *L. monocytogenes*, *B. subtilis*, *S. aureus* treated with SML increased by 2.7, 3.1, and 20.7 times, respectively (Figure 2d). The OD_260_ of supernatant from *L. monocytogenes*, *B. subtilis*, and *S. aureus* treated with SML were 1.5-, 1.7-, and 2.3-times as high as that of the control (Figure 2e). Similarly, the leakage of reducing sugar from *L. monocytogenes* and *B. subtilis* treated with SML increased by 54.7- and 4.3-times compared to the control, and *S. aureus* treated without SML even demonstrated the leakage of reducing sugar below the detection limit (Figure 2f). The above results indicated that damage to the CM did occur, which led to the losses of cell constituents and inhibition of cell growth.

### 3.5. Scanning Electron Microscopy (SEM) Analysis

The morphological changes of *L. monocytogenes*, *B. subtilis*, *S. aureus*, and *E. coli* were evaluated by SEM analysis. The scanning electron micrographs of both untreated and SML-treated bacterial cells are shown in Figure 3. Untreated cells showed regular and typical morphology, with a plump and smooth surface, and were uniform in size and distribution (Figure 3a,c,e,g). In contrast, *L. monocytogenes*, *B. subtilis*, and *S. aureus* cells treated with at 1× MIC concentration of SML (2.5 mM for *L. monocytogenes*, 2.5 mM for *B. subtilis*, and 10 mM for *S. aureus*) and revealed a severe damaging effect on the cell morphology, showing an irregularly wrinkled and coarse outer surface, the small leaking hole (Figure 3b,d,h) or even cell collapse could be observed (Figure 3f). This indicated that SML treatment may result in damage to the *L. monocytogenes, B. subtilis*, and *S. aureus* cell walls and CM. However, no obvious broken and rupture cells were found in *E. coli* cells treated with 40 mM SML, and only more wrinkled, coarse outer surface were observed by SEM at 20 k magnification (Figure 3h). The concentration for commercially available sugar esters used in foods and cosmetics as emulsifiers is up to 10 g L^−1^ [33], which is nearly twice as high as the MBC determined here for *L. monocytogenes* [25]. The MBCs for these tested organisms are greater than the critical micelle concentration (CMC) value for SML (0.2 to 0.8 mM) [34], indicating that SML concentrations greater than the CMC should be concerned to afford cell growth inhibition. Our results are consistent with other studies that evaluated the antimicrobial effects of various esters against Gram-positive bacteria. Similarly, it is proposed that monoglycerides as non-ionic surfactants penetrate and become combined with bacterial plasma membrane, thereby compromising membrane permeability. Some researchers suggested sucrose esters exerted their antibacterial effect through affecting the permeability of the cell membrane, resulting in the leakage of some cellular components such as proteins, reducing sugars [11].

### 3.6. Effects of SML on the Apparent Zeta Potential of Cells

**Figure 4:**
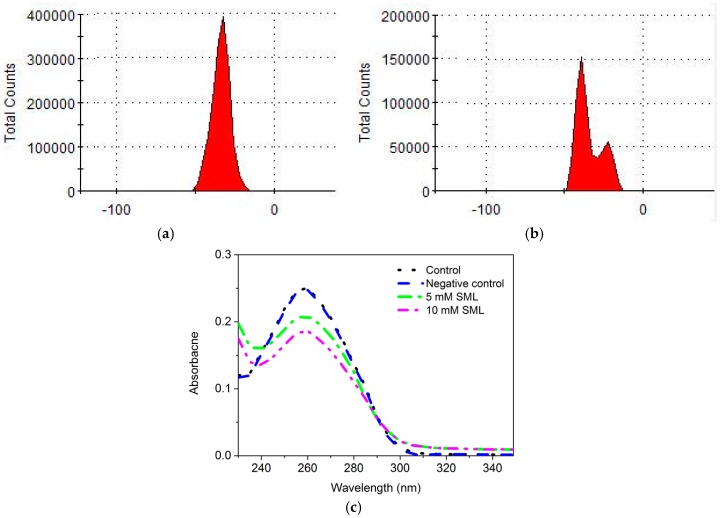
The effect of SML on zeta potential distribution of *L. monocytogenes*. (**a**) *L. monocytogenes*, without exposure to SML for 24 h; (**b**) *L. monocytogenes*, with exposure to SML for 24 h. Interaction of DNA of *L. monocytogents* with increasing amounts of SML; (**c**) Ultraviolet spectroscopic measurements.

The surface charge of cells is frequently determined based on their zeta potential, which is calculated from their electrophoretic motility in the presence of an electric field, under defined pH and ionic concentrations [35]. The time course of zeta potential measurements with *L. monocytogenes* and *E. coli* in the absence and presence of SML were presented in Figure S4. The alterations in zeta potential of *L. monocytogenes* and *E. coli* exhibited different patterns compared to their own controls. Bacterial cells normally present a negative surface charge, due to the presence of anionic groups in their membranes, such as carboxylate and phosphate groups [36]. Changes in the surface charge of both bacteria to less negative values were obtained after exposure to SML, indicating that cells treated with SML could to the depolarization of membrane. In addition, the release of K^+^ from the cytosol led to the alterations of the zeta potential of cells, which would disturb the subcellular localization of some proteins, and thereby cause the bacterial inactivation. For *L. monocytogenes,* where the effect of SML on the surface charge in zeta potential was more significant than that of *E. coli*. In comparison with the native control, less negative values were obtained when cells were exposed to SML (Figure S4a). In contrast to this, for *E. coli*, there are inconspicuous variation in zeta potential during the first 10 h regardless of exposure to SML (Figure S4b). Also, the obvious differences in zeta potential distribution of cells exist for such two representative bacteria, even though they were treated with the same conditions. A second peak located at apparent zeta potential of *L. monocytogenes* with treatment of SML was observed in Figure 4b, while this peak did not show when *L. monocytogenes* was treated without 10 mM SML for 24 h in Figure 4a. However, such a change did not occur in *E. coli* (Figure S4c,d). It could be inferred that there might be a different mode of action of SML for these two different bacteria.

### 3.7. Effect of SML on DNA of L. monocytogenes

The disruption of DNA, one of the most important genetic materials, could hinder gene expression, thereby leading to the restriction of normal enzyme and receptor synthesis, and inevitably inducing the death of bacteria. The interaction between SML and DNA, like the binding of it to DNA, was studied through ultraviolet and fluorescence spectra. As shown in Figure 4c, the addition of SML illustrated significant decrease of the maximum absorption intensity and a slight red shift, indicating the corresponding alternation in structure and conformation of DNA resulting from SML. SML may bind onto the phosphate group of DNA by hydrogen bonding, which could lead to reduction of the charge density in main chain and incensement the contraction of DNA structure [11]. The present study demonstrated that besides the cell wall and membrane, the bacterial genome might be another antibacterial target of SML, which fertilize the antibacterial mechanism of SML. Since SML showed binding effect with DNA, agarose gel electrophoresis of genomic DNA and DNA with increasing amounts of SML were used to evaluate whether it could induce the DNA damage. As shown in Figure S5, the bands of DNA treated with different concentrations of SML were similar to that of control, which indicated SML had no ability to cause significant DNA damage. These results are in accordance with the findings obtained by Zhao et al. [11].

### 3.8. Effect of SML at Sub-MICs on the Inhibition of Biofilm Formation

**Figure 5:**
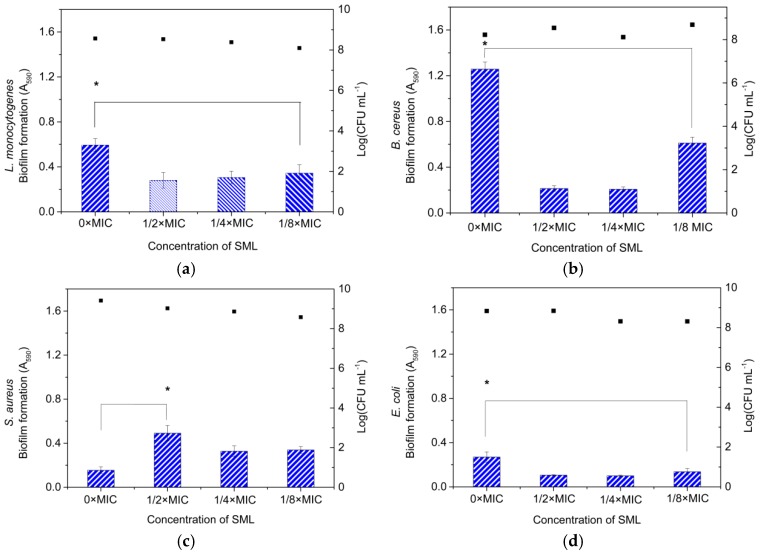
Effect of different concentrations of SML on the biofilm formation of (**a**) *L. monocytogenes*; (**b**) *B. subtilis*; (**c**) *S. aureus*; (**d**) *E. coli*. The error bars represent the standard deviations, and the asterisks indicate significant difference between each other.

SML is completely safe and if it had biofilm inhibitory activity, it would be suitable to be employed in controlling biofilms in food applications. Microbial biofilms represent a distinct bacterial physiology characterized by a multicellular phenotype that is fundamentally different from planktonic bacteria. In this section, we explored the differing effects of SML at sub-MICs on Gram-positive and Gram-negative bacterial strains. Figure 5 showed that SML exhibits the specific ability for inhibition of biofilm formation by these bacteria except for *S. aureus*. SML could significantly inhibit the biofilm formation by *B. subtilis*, *L. monocytogenes*, and *E. coli*. The degree of inhibition of biofilm formation resulted from SML was relatively concentration-dependent. Compared to the control, the biofilm formation from *L. monocytogenes*, *B. subtilis*, and *E. coli* treated with 1/4× MIC SML reduced by 1.9-, 6.1-, and 2.6-times, respectively, demonstrating that the efficacy of SML to spoil the biofilm formation by *B. subtilis* is the most remarkable among them. We speculated that the physical factors such as nature of electric charge of the cell surface or physical interaction between cell and solid surface might be of more importance [35].

In contrast, the biofilm formation by *S. aureus* was stimulated by SML at sub-MICs, which could be rationally used to explain why *S. aureus* is more inherently resistant to SML than other Gram-positive bacteria in Figure S2. This was also found in recent experiments on biofilm formation, where all the antibiotics tested were effective in laboratory MIC tests but lost their efficacy against *S. aureus* biofilms in sub-MICs [37]. On the other hand, the growth of these four bacteria was mostly unaffected at sub-MICs (<1/2× MIC), which is also in agreement with the results obtained in Figure S2. It is also speculated that the inhibition of SML to them is related not only to physicochemical properties of cell surface such as chemical structure and composition or nature of electric charge of the cell surface but also to chemical interaction between cells/biofilm and antibacterial compounds or physical interaction between cells and solid surface [35].

## 4. Conclusions

This work investigated the antibacterial properties of enzymatically synthesized SML against four pathogenic bacteria including *L. monocytogenes*, *B. subtilis*, *S. aureus*, and *E. coli*. SML could exhibit significant antimicrobial activity against tested bacterial and anti-biofilm formation for these bacteria, especially against Gram-positive bacteria. The potential antibacterial mechanisms of SML were related to permeability and integrity of cell envelops, resulting in leakage of some cellular components (proteins, reducing sugars, 260 nm absorbing materials and K^+^, as well as β-galactosidase). Similarly, SML might bind with DNA to hinder DNA replication. Moreover, SML at sub-MIC significantly inhibited biofilm formation by *L. monocytogenes*, *B. subtilis*, and *E. coli*, especially for *B. subtilis*, whereas their inhibitory effect was invalid in the case of *S. aureus* biofilm formation. More broadly, we expect this study’s ability to find the use of other biosurfactants on their own or as adjuvants may represent a potential way forward in tackling foodborne infections and biofilms in food processing environments in the future.

## Supplementary Materials

The supplementary materials are available online.
