# Supplementary material for: Lipase-Catalyzed Synthesis of Sucrose Monolaurate and Its Antibacterial Property and Mode of Action against Four Pathogenic Bacteria
Source: Molecules. 2018 May 8;23(5):1118. doi: 10.3390/molecules23051118 (PMC6100556; doi:10.3390/molecules23051118)
Supplement: Supplementary file 1 [file molecules-23-01118-s001.pdf]

molecules

MDPI

Article

Lipase-catalyzed synthesis of sucrose monolaurate and its antibacterial property and mode of action against four pathogenic bacteria

Shi-yin Shao [a, b], Yu-gang Shi [a, b]*Yu Wu [a, b], Li-qing Bian [a, b], Yun-jie Zhu [a, b], Xin-ying Huang [a, b], Ying Pan [a, b], Lu-yao Zeng [a, b], Run-run Zhang [a, b]

[1] Zhejiang Provincial Collaborative Innovation Center of Food Safety and Nutrition, Zhejiang Gongshang University, Hangzhou, Zhejiang 310035, China

[2] School of Food Science and Biotechnology, Zhejiang Gongshang University, Hangzhou, Zhejiang 310035, China

* Correspondence: yugangshi@zjgsu.edu.cn; Tel.: +86–0571–28008927

Received: date; Accepted: date; Published: date

Supporting Information

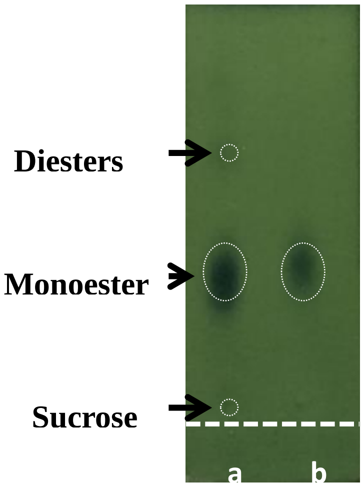

(a)

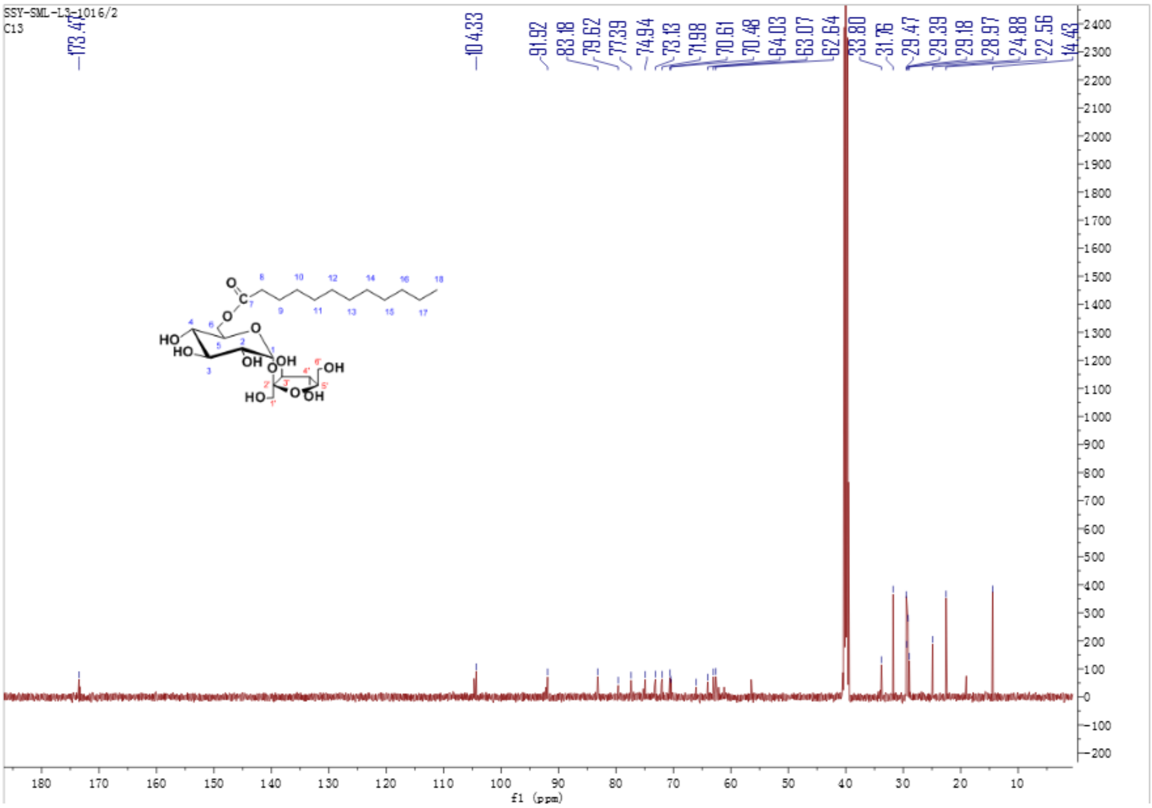

(b)

Figure S1: (a) Enzymatic catalyzed sucrose ester synthesis in novel functionalized ionic liquid. TLC analysis of reactions: (line a) Before purification. (line b) After purification ; (b) The 13C NMR spectrum of the enzymatically synthesized SML.

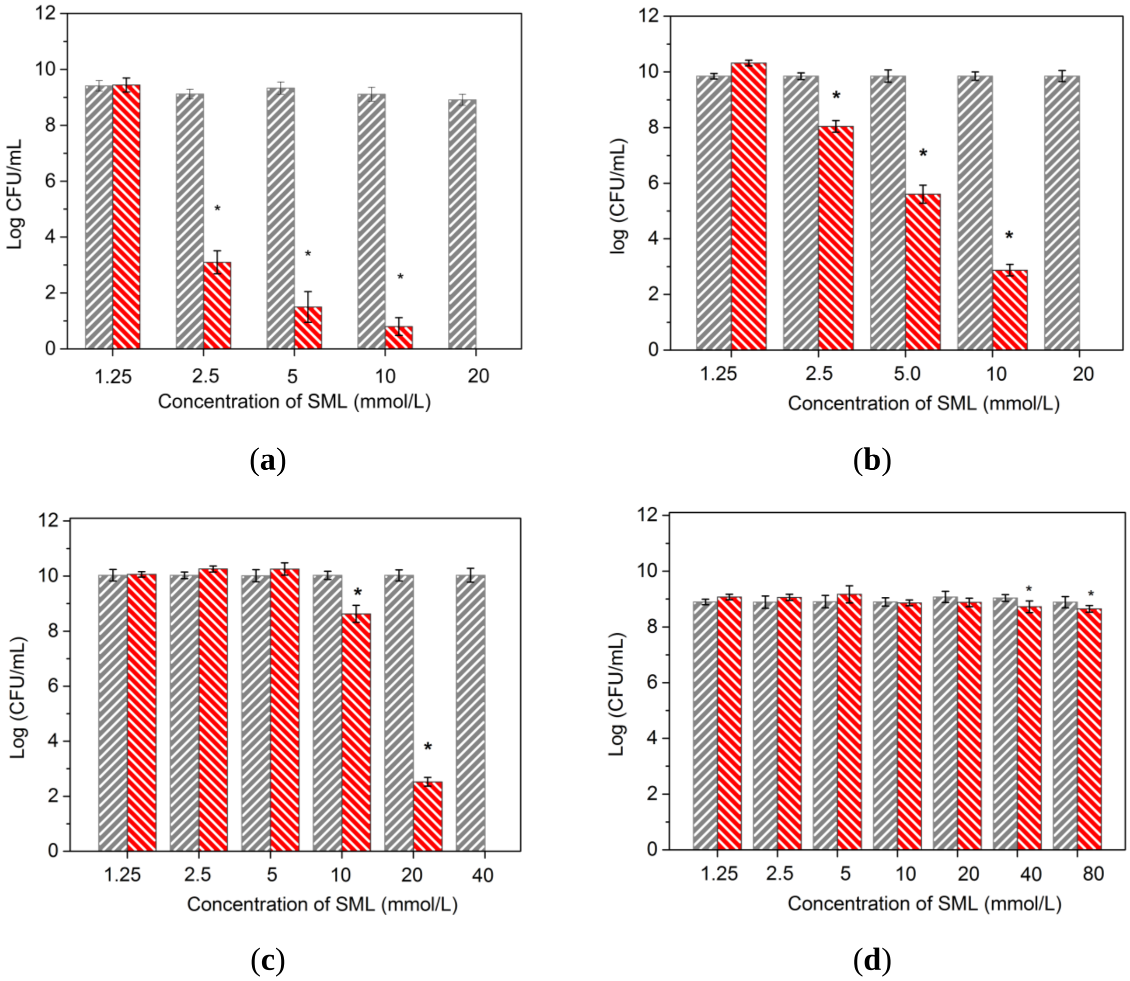

Figure S2: Average counts (log CFU mL^{-1}) of various microorganisms treated with 1.25, 2.5, 5.0, 10, 20, 40 and 80 SML. (a) *L. monocytogenes*; (b) *B. subtilis*; (c) *S. aureus*; (d) *E. coli*. The error bars represent the standard deviations, and the asterisks indicate significant difference between each other.

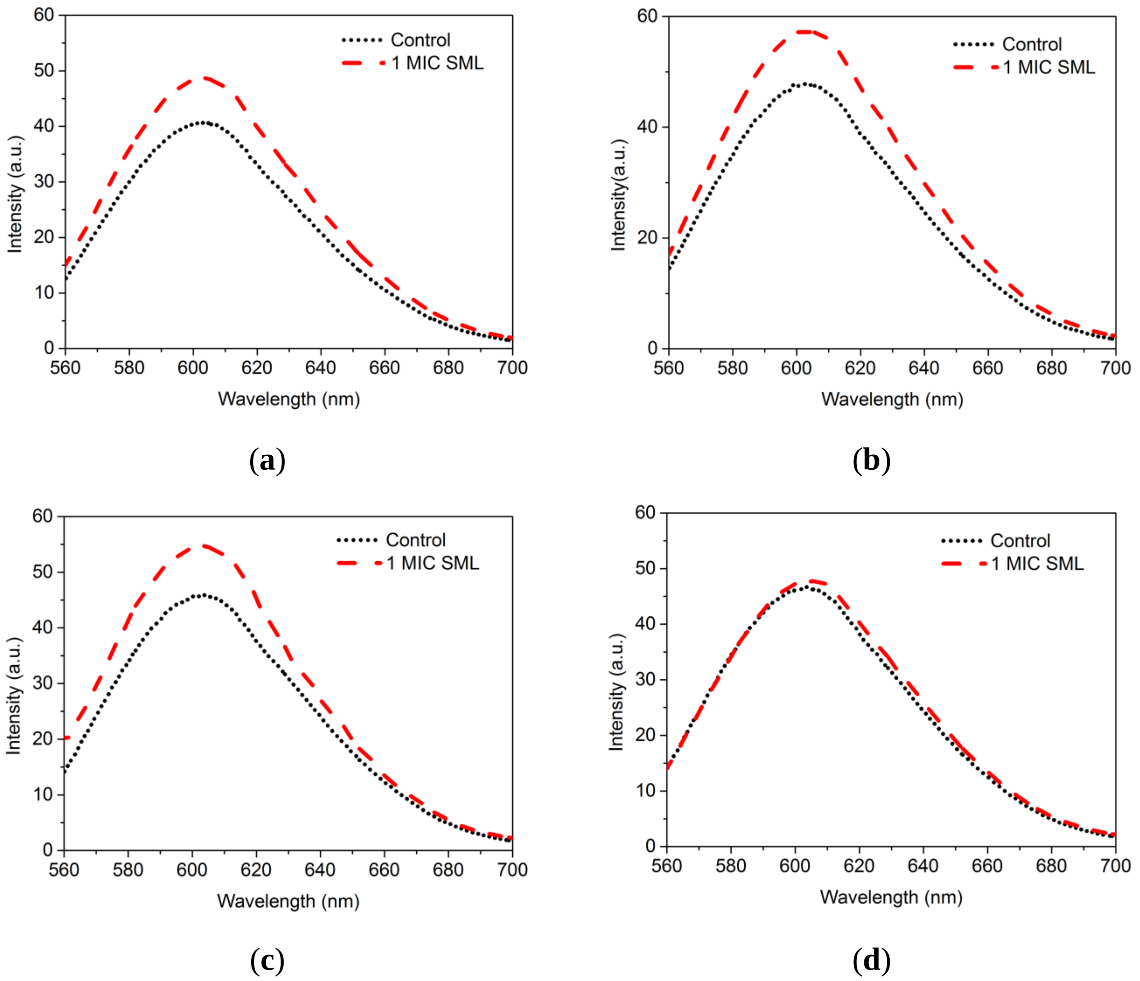

Figure S3: The fluorescence spectra of PI in cells treated with 1× MIC SML for 6 h. (a) *L. monocytogenes*; (b) *B. subtilis*; (c) *S. aureus*; (d) *E. coli*.

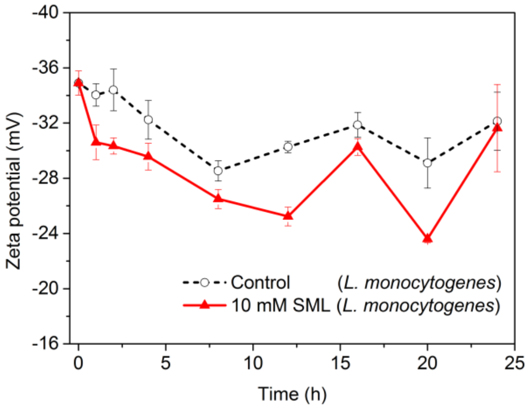

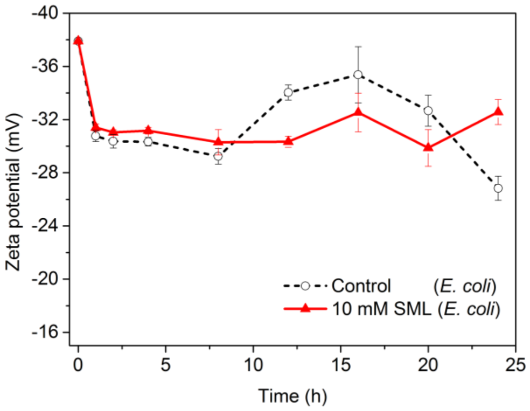

(a) **(b)**

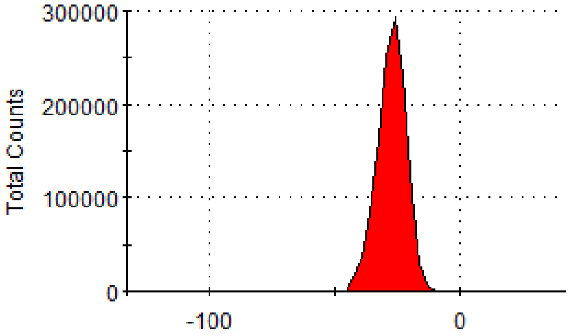

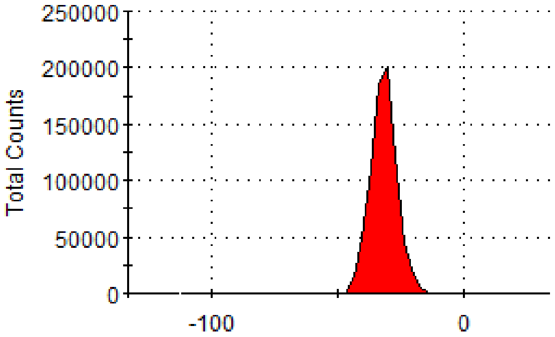

(c) **(d)**

Figure S4: The effect to SML on zeta potential of *L. monocytogenes* (a) and *E. coli* (b).The effect of SML on zeta potential distribution of *E. coli*; (c) *E. coli*, without exposure to SML for 24 h. (d) *E. coli*, with exposure to SML for 24 h.

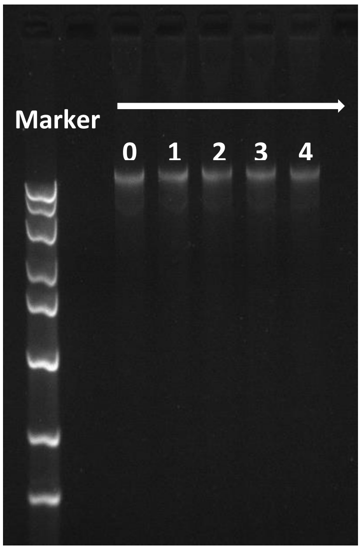

Figure S5: Electrophoresis map of DNA extracted from *L. monocytogents* treated with different concentrations of SML. Lane 0: control; Lane 1: negative control; and Lines 2-4: 5 mM, 10 mM and 20 mM of SML.
